# Neural Substrates of Semantic Prospection – Evidence from the Dementias

**DOI:** 10.3389/fnbeh.2016.00096

**Published:** 2016-05-24

**Authors:** Muireann Irish, Nadine Eyre, Nadene Dermody, Claire O’Callaghan, John R. Hodges, Michael Hornberger, Olivier Piguet

**Affiliations:** ^1^Neuroscience Research Australia, Sydney, NSWAustralia; ^2^School of Psychology, The University of New South Wales, Sydney, NSWAustralia; ^3^Australian Research Council Centre of Excellence in Cognition and its Disorders, Sydney, NSWAustralia; ^4^Behavioural and Clinical Neuroscience Institute, University of Cambridge, CambridgeUK; ^5^Brain and Mind Centre, The University of Sydney, Sydney, NSWAustralia; ^6^School of Medical Sciences, The University of New South Wales, Sydney, NSWAustralia; ^7^Norwich Medical School, University of East Anglia, NorwichUK

**Keywords:** episodic memory, semantic memory, imagination, hippocampus, prefrontal cortex, Alzheimer’s disease, frontotemporal dementia, future thinking

## Abstract

The ability to envisage personally relevant events at a future time point represents an incredibly sophisticated cognitive endeavor and one that appears to be intimately linked to episodic memory integrity. Far less is known regarding the neurocognitive mechanisms underpinning the capacity to envisage non-personal future occurrences, known as semantic future thinking. Moreover the degree of overlap between the neural substrates supporting episodic and semantic forms of prospection remains unclear. To this end, we sought to investigate the capacity for episodic and semantic future thinking in Alzheimer’s disease (*n* = 15) and disease-matched behavioral-variant frontotemporal dementia (*n* = 15), neurodegenerative disorders characterized by significant medial temporal lobe (MTL) and frontal pathology. Participants completed an assessment of past and future thinking across personal (episodic) and non-personal (semantic) domains, as part of a larger neuropsychological battery investigating episodic and semantic processing, and their performance was contrasted with 20 age- and education-matched healthy older Controls. Participants underwent whole-brain T_1_-weighted structural imaging and voxel-based morphometry analysis was conducted to determine the relationship between gray matter integrity and episodic and semantic future thinking. Relative to Controls, both patient groups displayed marked future thinking impairments, extending across episodic and semantic domains. Analyses of covariance revealed that while episodic future thinking deficits could be explained solely in terms of episodic memory proficiency, semantic prospection deficits reflected the interplay between episodic and semantic processing. Distinct neural correlates emerged for each form of future simulation with differential involvement of prefrontal, lateral temporal, and medial temporal regions. Notably, the hippocampus was implicated irrespective of future thinking domain, with the suggestion of lateralization effects depending on the type of information being simulated. Whereas episodic future thinking related to right hippocampal integrity, semantic future thinking was found to relate to left hippocampal integrity. Our findings support previous observations of significant MTL involvement for semantic forms of prospection and point to distinct neurocognitive mechanisms which must be functional to support future-oriented forms of thought across personal and non-personal contexts.

## Introduction

The ability to mentally project oneself across past and future contexts is a highly sophisticated cognitive capacity, which confers incredible flexibility in our daily lives. Prospection, or future-oriented mental time travel, represents a source of intense research effort with a dramatic surge in studies seeking to elucidate the cognitive and neural architecture of this complex ability. Functional neuroimaging studies in healthy individuals converge to reveal striking overlap in the brain regions that support episodic retrieval of the past and those which underpin simulation of future events (Addis et al., 2007; Szpunar et al., 2007; Botzung et al., 2008), suggestive of a common neural substrate subtending both forms of thought. Moreover, studies of clinical populations characterized by episodic memory dysfunction reveal parallel deficits irrespective of temporal context, further supporting a close coupling between episodic memory integrity and future simulation (Hassabis et al., 2007; Andelman et al., 2010; Race et al., 2011; but see Squire et al., 2010).

Not surprisingly, future thinking has tended to be couched within the episodic memory system and the vast majority of studies conducted to date have focused on episodic forms of prospection (reviewed by Klein, 2013a). The capacity to engage in future-oriented forms of thinking, however, relies upon many additional component processes beyond episodic memory, including mental imagery, fluency, specificity, and phenomenological processes such as introspection and the apprehension of subjective time (D’Argembeau et al., 2010). Moreover, studies of future thinking in clinical populations have revealed the importance of semantic memory for temporal (Duval et al., 2012; Irish et al., 2012a,b) and atemporal (Cooper et al., 2011) forms of imagination. These findings have led to the advancement of the semantic scaffolding hypothesis which holds that semantic memory may not only facilitate, but may be essential for, future oriented thought (Irish and Piguet, 2013).

While evidence is accruing regarding the role of semantic memory in supporting episodic future thinking, far less is known regarding the converse relationship, i.e., whether episodic memory is crucial for semantic prospection. The majority of studies to date have focused on episodic future thinking; however, it is clear that prospection does not reside exclusively in the episodic domain (Klein, 2013a). For example, humans can contemplate past world events to make informed inferences regarding potential non-personal future occurrences such as advances in medicine or politics. In recognition of this distinction, Abraham et al. (2008) investigated the neural substrates of past and future thinking using fMRI in healthy young individuals and revealed dissociations across personal (episodic) and non-personal (semantic) contexts. Medial temporal lobe (MTL) activity was identified across personal and non-personal future conditions, suggesting that episodic and semantic forms of prospection recruit similar underlying processes subserved by the hippocampus (Race et al., 2013).

Insights into the neurocognitive mechanisms supporting semantic forms of future thinking have also emerged from the study of amnesic populations. Patient D.B. suffered a global loss of anterograde and retrograde episodic memory manifesting in severe impairments in remembering his personal past and constructing his personal future. Despite these difficulties in the episodic domain, D.B. displayed a relatively preserved capacity to retrieve past non-personal and to envisage non-personal future issues (Klein et al., 2002), suggesting that non-episodic memory can facilitate future thinking in the absence of episodic content (see also Kwan et al., 2012). More recently, we demonstrated gross semantic prospection impairments in patients with semantic dementia (Irish et al., 2012a). These deficits were attributable to degeneration of left anterior temporal regions known to support semantic processing, underscoring a clear link between general conceptual processing and simulation of semantic content. Non-personal future thinking has also been found to be impaired in a patient who underwent left anteromedial temporal lobe resection to control his epilepsy, with these deficits suggested to stem largely from a selective impairment of public semantic memory (Manning et al., 2013). Collectively, these studies suggest that general semantic processing is a prerequisite for non-personal forms of future thinking (reviewed by Irish, 2016).

Notably, however, a recent study has highlighted the potential interplay between episodic memory processes subserved by the MTLs and semantic forms of prospection (Race et al., 2013). Hippocampal amnesics performed at control levels when generating semantic facts about the future, yet when probed to elaborate upon these facts, patients displayed a paucity of detail. Importantly, this compromised capacity to elaborate upon semantic future issues occurred independently of general conceptual knowledge as the patients displayed intact performance on neuropsychological tests of semantic processing. Accordingly, it was proposed that the MTLs support prospection across episodic and semantic domains potentially by disrupting detail generation and associative binding (Schacter and Addis, 2009).

To date, only a handful of studies have investigated non-episodic forms of future thinking in clinical populations, and it remains unclear whether semantic prospection recruits similar or largely distinct neurocognitive processes as compared to episodic prospection. The current study sought to determine the neurocognitive mechanisms underpinning semantic prospection by investigating past and future thinking performance across episodic and semantic domains in two neurodegenerative disorders; Alzheimer’s disease (AD) and the behavioral-variant of frontotemporal dementia (bvFTD). Both patient groups are characterized by significant impairments in episodic encoding and retrieval (Hornberger et al., 2012; Frisch et al., 2013; Irish et al., 2014), autobiographical memory retrieval (Piolino et al., 2003; Irish et al., 2011), and episodic future thinking (Irish et al., 2013; reviewed by Irish and Piolino, 2016), in the context of variable impairments in semantic memory (reviewed by Karantoulis, 2011). An important outstanding question, however, is whether these patients are capable of envisaging non-personal or semantic future issues and the neurocognitive mechanisms that must be functional to support this form of prospection. To investigate this question we administered the Memory and Temporal Experience questionnaire developed by Klein et al. (2002) as part of a larger neuropsychological battery investigating aspects of episodic and semantic processing. This approach would allow us to determine how integrity of episodic and semantic processing impacts the capacity for future thinking in the semantic domain.

## Materials and Methods

### Participants

A total of fifty participants were included in this study. Fifteen individuals meeting current clinical diagnostic criteria for behavioral variant frontotemporal dementia (bvFTD; Rascovsky et al., 2011) and fifteen individuals diagnosed with clinically probable AD (McKhann et al., 2011) were recruited through FRONTIER, the frontotemporal dementia clinic at Neuroscience Research Australia (NeuRA) in Sydney. Clinical diagnosis was established by multidisciplinary consensus among neurologist, neuropsychologist, and occupational therapist based on extensive clinical investigations, cognitive assessment, informant interviews, and evidence of atrophy on structural neuroimaging. Briefly, bvFTD patients presented with marked changes in behavior and personality, executive dysfunction, and socioemotional dysregulation. In contrast, AD patients displayed significant episodic memory dysfunction, disorientation to time and place, and visuospatial deficits, in the context of relatively preserved emotion processing and comportment.

Twenty healthy older control participants were recruited from local community groups and the NeuRA volunteer research panel. All controls scored 0 on the Clinical Dementia Rating scale (CDR; Morris, 1997) and 88 or above on the Addenbrooke’s Cognitive Examination-Revised (ACE-R; Mioshi et al., 2006). Exclusion criteria for all participants included prior history of mental illness, significant head injury, movement disorders, cerebrovascular disease, alcohol and other drug abuse, and limited English proficiency.

### Neuroimaging

All participants underwent whole-brain T_1_-weighted imaging using a 3T Philips MRI scanner with standard quadrature head coil (eight channels) using the following sequences: coronal orientation, matrix 256 × 256, 200 slices, 1 mm^2^ in-plane resolution, slice thickness 1 mm, echo time/repetition time = 2.6/5.8 ms, flip angle α = 19°. A structural scan was not available for one control participant. All scans were examined by a neuroradiologist for structural abnormalities; none were reported for control participants. AD patients displayed characteristic MTL atrophy involving the hippocampus bilaterally, in the context of frontal and parietal atrophy. BvFTD patients displayed significant prefrontal and anteromedial temporal lobe atrophy including the hippocampus bilaterally.

### Ethical Approval

This study was conducted in accordance with the Declaration of Helsinki. Ethical approval was obtained from the Human Research Ethics Committee of the South Eastern Sydney and Illawarra Area Health Service (HREC 10/126) and the University of New South Wales Human Research Ethics Advisory panel D (Biomedical, ref. # 10035). All participants, or their person responsible, provided written informed consent. Capacity to provide informed consent was established by asking participants to signify that they understood the purpose of the research visit by explaining the proposed research in their own words. In the event that patients lacked the capacity to provide informed consent, written informed consent was obtained from the patient’s next of kin or legally authorized representative. Withdrawal from the study was permitted at any time if either the patient or the family member elected to discontinue. Participants volunteered their time and were reimbursed for travel costs.

### General Cognitive Assessment

Participants completed a comprehensive battery of neuropsychological tests. The ACE-R was used as a general measure of overall cognitive functioning assessing attention and orientation, memory, fluency, language, and visuospatial function (Mioshi et al., 2006). Episodic memory integrity was assessed across verbal and non-verbal domains. Delayed verbal episodic recall was measured using the Rey Auditory Verbal Learning Task (RAVLT; Schmidt, 1996). The 3-min recall of the Rey Complex Figure (RCF; Rey, 1941) was used as an index of non-verbal episodic retrieval. A percentage retained score was computed by dividing the RCF recall score by the RCF Copy score (i.e., Recall/Copy*100). Semantic processing was assessed using verbal letter fluency (F,A,S; Strauss et al., 2006) and the Naming, Comprehension, and Semantic Association subscales of the Sydney Language Battery (SydBAT; Savage et al., 2013).

In addition, psychomotor speed and mental flexibility were measured using the Trail Making Test Parts A and B (Reitan, 1958), respectively. A Trails B–A difference score was computed to reflect the capacity for set-switching and divided attention. Basic attention and working memory was assessed using Digit Span forward and backward, respectively (Wechsler, 1997). Finally, the functional status of patients was determined using the Frontotemporal Dementia Rating Scale (FRS; Mioshi et al., 2010) which is a dementia staging tool sensitive to changes in functional ability.

### Assessment of Past and Future Thinking

#### Episodic Past and Future

The Memory and Temporal Experience Questionnaire (Klein et al., 2002) was used to explore the capacity for past and future thinking across “Lived” (episodic) and “Known” (semantic) conditions. Briefly, participants completed a series of 10 questions requiring the recollection of personally relevant past events over the previous year (Lived Past, e.g., “What did you do yesterday?”). In the corresponding Lived Future condition, the same 10 questions were administered, matched for temporal displacement, but situated within the next year (e.g., “What will you do tomorrow?”). Participants were required to speak extemporaneously in response to each question, however, general prompts were provided (e.g., “Can you tell me any more details about this?”). No time limit was imposed for responding. Responses were awarded a maximum of 3 points for each question contingent on the provision of event (1 point), place (1 point), and time (1 point) details, corresponding to the “what/where/when” of episodic memory. Responses were coded as 0 for false/irrelevant answers or the failure to provide any information. As such, the maximum score for each temporal condition was 30 points (i.e., 10 questions × 3 points). While none of the patients in this study had a documented history of confabulation, we nevertheless cross-checked improbable responses with informants to ensure veracity.

#### Semantic Past and Future

The “Known” subscale of the Memory and Temporal Experience Questionnaire was used to probe semantic information across past and future contexts. Participants were asked seven questions regarding non-personal public events and issues that took place over the preceding 10 years and that could potentially occur within the next 10 years. This time period was specifically chosen to ensure that the material being provided was not contaminated by episodic retrieval. The domains covered included politics, community issues, national issues, medical breakthroughs, issues for the planet, advances in technology, and environmental issues. The questions across past and future contexts differed only with respect to the temporal orientation. For example, “Can you tell me what have been some of the most important political events occurring over the last 10 years” (Semantic Past), and “Can you tell me what you think will be some of the most important political events in the next 10 years?” (Semantic Future). Responses were scored in terms of plausibility and level of detail, receiving 0 points (no information/implausible response), 1 point (provides one plausible example) and 2 points (provides at least two plausible and detailed examples). As such, the maximum score for each temporal condition was 14 points (i.e., 7 questions × 2 points). Given that we wished to compare performance across the Episodic and Semantic conditions, all scores were scaled as a proportion of the total score for each domain (e.g., Episodic Past/30*100; Semantic Past/14*100).

Order was counterbalanced across Episodic and Semantic conditions to guard against order effects. All interview transcripts were scored by NE. A subset of transcripts were randomly selected from fifteen participants (five AD, five bvFTD, five Control) and scored by ND, blind to diagnosis and study objectives. Inter-rater reliability was calculated using the intra-class correlation coefficient and revealed excellent convergence between the two raters across all subscales (Episodic Past: α = 0.986; Episodic Future, α = 0.984; Semantic Past, α = 0.955; Semantic Future, α = 0.970).

### Statistical Analyses

Cognitive data were analyzed using IBM SPSS Statistics (Version 22). Multivariate analyses of variance (MANOVA) were run to investigate main effects of group (AD, bvFTD, Controls) across each of the background neuropsychological tests. For the experimental task, mixed-model ANCOVAs, with age included as a covariate, were run to explore main effects of time (Past, Future) and domain (Episodic, Semantic). Sidak *post hoc* tests were used to explore main effects of group for all variables of interest. Pearson R correlations were run to investigate potential associations between past and future thinking performance and neuropsychological tests of interest. Finally, Chi-squared tests (*X*^2^), based on the frequency patterns of dichotomous variables (e.g., sex), were used.

### Voxel-Based Morphometry Analyses

Structural MRI data were analyzed with FSL-VBM, a voxel-based morphometry (VBM) analysis (Ashburner and Friston, 2000; Mechelli et al., 2005) using the FSL-VBM toolbox from the FMRIB software package^1^ (Smith et al., 2004). Structural images were extracted using the FSL brain extraction tool (BET; Smith, 2002), following which, tissue segmentation was carried out using FMRIB’s Automatic Segmentation Tool (FAST; Zhang et al., 2001). Gray matter partial volumes were then aligned to the Montreal Neurological Institute standard space (MNI152) using the FMRIB non-linear registration approach (FNIRT; Andersson et al., 2007a,b) using a b-spline representation of the registration warp field (Rueckert et al., 1999). A study-specific template was then created to which the native gray matter images were re-registered non-linearly. The registered partial volume maps were modulated by dividing by the Jacobian of the warp field, to correct for local expansion or contraction. Finally, the modulated segmented images were smoothed with an isotropic Gaussian kernel with a sigma of 3 mm.

A whole-brain voxel-wise general linear model was applied to investigate gray matter intensity differences via permutation-based non-parametric testing (Nichols and Holmes, 2002) with 5000 permutations per contrast. First, differences in cortical gray matter intensities between patients (AD or bvFTD) and Controls were assessed. Clusters from the group atrophy analyses were extracted using the threshold-free cluster enhancement method (tfce) and corrected for Family-Wise Error (FWE) at *p* < 0.05.

Next, correlations between Episodic and Semantic Future thinking performance and gray matter intensity were investigated using a whole-brain approach in the AD and bvFTD patients combined. This approach was adopted to increase the statistical power to detect brain-behavior relationships across the entire brain by achieving greater variance in behavioral scores. For statistical power, a covariate only statistical model with a positive *t*-contrast was used, providing an index of association between gray matter intensity and future thinking performance. Clusters were extracted using a voxel-wise approach and corrected for False Discovery Rate at *p* < 0.05. Age was included as a nuisance variable in the atrophy and covariate analyses. In addition, the corresponding past retrieval condition was included as a covariate in the model to control for common neural substrates implicated across past and future conditions. Two models were therefore run investigating (i) Episodic Future thinking controlling for Episodic Past retrieval and Age (1, 0, 0) and (ii) Semantic Future thinking controlling for Semantic Past retrieval and Age (1, 0, 0). Anatomical locations of significant results were overlaid on the MNI standard brain, with maximum coordinates provided in MNI stereotaxic space. Anatomical labels were determined with reference to the Harvard–Oxford probabilistic cortical atlas.

## Results

### Demographics

The participant groups did not differ significantly in terms of age (*p* = 0.360), years in education (*p* = 0.211), or sex distribution (*p* = 0.603; **Table 1**). In addition, bvFTD and AD patient groups were matched for disease duration (years elapsed since onset of symptoms, *p* = 0.668) and overall level of cognitive functioning on the ACE-R (*p* = 0.130), however, bvFTD patients showed significantly higher overall levels of functional impairment relative to AD patients (FRS: *p* = 0.003).

**Table 1:** Demographic and clinical characteristics of study samples^a,b,c^.

	AD (*n* = 15)	bvFTD (*n* = 15)	Controls (*n* = 20)	Group effect	*Post hoc* test
Sex (M:F)	10:5	9:6	10:10	n/s	–
Age (years)	65.4 (7.7)	63.5 (7.4)	67.1 (7.0)	n/s	–
Education (years)	11.9 (3.7)	11.6 (3.0)	13.3 (2.0)	n/s	–
Disease duration (years)	4.3 (2.5)	3.8 (2.6)	–	n/s	–
FRS Rasch logit score	1.3 (1.2)	–0.4 (1.5)	–	**	bvFTD < AD
ACE-R total (100)	74.7 (8.6)	79.4 (6.7)	94.5 (3.3)	***	Patients < Controls
RCF copy (36)	23.8 (10.3)	26.7 (5.8)	31.1 (4.3)	*	AD < bvFTD, Controls
RCF recall (% retained)	24.4 (21.8)	19.3 (14.0)	52.0 (16.3)	***	Patients < Controls
RAVLT 30 min recall (15)	2.7 (2.8)	4.1 (3.6)	10.6 (2.8)	***	Patients < Controls
Trails Part A (sec)	53.9 (29.7)	49.0 (20.6)	34.8 (10.1)	n/s	–
Trails Part B–A (sec)	110.2 (59.3)	65.9 (28.2)	51.5 (22.5)	**	AD > bvFTD, Controls
Digit span forward	5.9 (1.0)	6.1 (1.3)	7.2 (1.2)	**	Patients < Controls
Digit span backward	4.0 (0.8)	3.5 (1.0)	5.3 (1.4)	***	Patients < Controls
Letter fluency total	29.9 (14.7)	17.8 (6.9)	48.6 (14.2)	***	bvFTD < AD < Controls
Naming (30)	21.3 (4.2)	21.3 (3.4)	26.7 (2.4)	***	Patients < Controls
Comprehension (30)	26.1 (2.2)	26.7 (1.7)	28.9 (1.6)	***	Patients < Controls
Semantic association (30)	25.2 (3.0)	23.6 (3.4)	28.1 (1.8)	***	Patients < Controls

*^a^Maximum score for each test and standard deviations in brackets where applicable.*

*^b^bvFTD, behavioral variant frontotemporal dementia; AD, Alzheimer’s disease; FRS, Frontotemporal Dementia Rating Scale; ACE-R, Addenbrooke’s Cognitive Examination Revised; RCF, Rey Complex Figure test; RAVLT, Rey Auditory Verbal Learning Test.*

^c^FRS Rasch logit scores available for 14 AD cases. Trail Making Test data available for 19 Controls. Trail Making Test A discontinued in two AD cases. Trail Making Test B discontinued in five bvFTD and seven AD cases. RAVLT data available for 12 AD cases and 19 Controls; Digit span and letter fluency data available for 19 Controls, SydBAT data available for 18 Controls.

*p < 0.05; **p < 0.01; ***p < 0.0001; n/s, non-significant; ‘–’, not applicable.

### General Cognitive Functioning

Neuropsychological testing revealed characteristic profiles of deficits in the bvFTD and AD groups relative to Controls (**Table 1**). Both patient groups displayed significant declines in global cognitive functioning as measured by the ACE-R (all *p*-values <0.0001) with no significant difference between the patient groups (*p* = 0.130). Delayed episodic memory performance was significantly compromised across verbal and non-verbal indices in both patient groups relative to Controls (all *p*-values <0.0001) with AD and bvFTD patients showing comparable performance (*p* = 0.585). Semantic processing was also significantly impaired with deficits evident across Naming (all *p*-values <0.0001) Comprehension (AD: *p* < 0.0001; bvFTD: *p* = 0.004), and Semantic Association (AD: *p* = 0.013; bvFTD: *p* < 0.0001) subscales of the SydBAT, and no significant differences between the patient groups (all *p*-values >0.3). Speed of processing was relatively intact in both AD and bvFTD relative to Controls (all *p*-values >0.3). Set-shifting was significantly compromised in the AD group relative to Controls (*p* = 0.001) and bvFTD patients (*p* = 0.032), whereas bvFTD patients scored in line with Controls (*p* = 0.652). Impairments in verbal working memory were observed relative to Controls (Digit span backward: AD, *p* = 0.004; bvFTD, *p* < 0.0001) with no significant differences between the patient groups (all *p*-values >0.5). Verbal letter fluency was also significantly compromised in AD and bvFTD (all *p*-values <0.0001) with AD scoring significantly higher than bvFTD patients (*p* = 0.036). Finally, impairments in visuospatial processing on the RCF Copy were observed in AD (*p* = 0.011) but not in bvFTD (*p* = 0.197) as compared with Controls, with no difference between the patient groups (*p* = 0.608).

### Past and Future Thinking Performance

**FIGURE 1:**
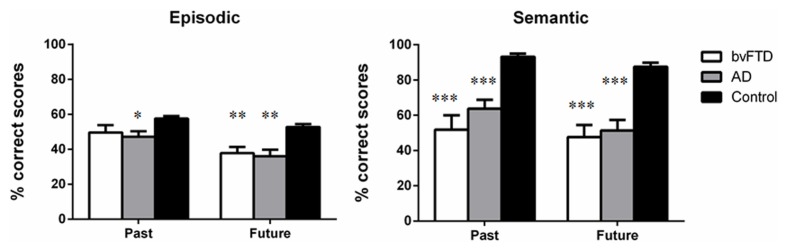
**Past and future thinking performance across episodic and semantic domains for behavioral-variant frontotemporal dementia (bvFTD), Alzheimer’s disease (AD), and Control participants.** Scores represent percentage correct performance for each subscale. Error bars represent standard error of the mean. Asterisks denote group differences relative to Controls. *p < 0.05; **p < 0.01; ***p < 0.0001.

**Figure 1** illustrates past and future thinking performance across episodic and semantic domains for all participant groups. A mixed-model multivariate ANCOVA, with age as a covariate, revealed an overall main effect of Group [*F*(2,46) = 21.046, *p* < 0.0001]. Sidak *post hoc* tests confirmed that patients were significantly compromised, irrespective of domain or temporal context, relative to Controls (bvFTD; *p* < 0.0001; AD, *p* < 0.0001). No significant differences were evident between the patient groups (*p* = 0.857).

A significant main effect of Time was found [*F*(1,46) = 4.037, *p* = 0.050] with Past performance significantly higher than Future, irrespective of domain (*p* < 0.0001). A significant main effect of Domain was also observed [*F*(1,46) = 4.569, *p* = 0.038] reflecting the fact that semantic performance was significantly higher than episodic performance, irrespective of temporal context (*p* < 0.0001).

Finally, a significant Group × Domain interaction was evident [*F*(2,46) = 18.627, *p* < 0.0001]. This interaction reflected the fact that for episodic information Controls scored significantly higher than bvFTD (*p* = 0.006) and AD (*p* = 0.001) participants, with no significant difference between the patient groups (*p* = 0.959). For semantic information, the same profile of results was obtained with Controls outperforming both patient groups (all *p*-values <0.0001) and no significant differences between the patient groups (*p* = 0.507).

Within group comparisons further revealed higher levels of performance in the semantic versus episodic domain for AD (*p* < 0.0001) and Control (*p* < 0.0001) participants, however, this effect was not observed in the bvFTD group (*p* = 0.163).

No other significant interactions were evident (all *p*-values >0.1).

### Correlations between Future Thinking Performance and Neuropsychological Variables

Pearson R correlations are displayed in **Table 2**. We first investigated the relationship between past retrieval and future thinking performance on the experimental task.

**Table 2:** Pearson R correlations between future thinking performance and episodic and semantic domains in bvFTD and AD participant groups.

Group		Episodic	Semantic
		Future	Future
bvFTD	Episodic Past	0.709**	0.632*
	Semantic Past	0.658**	0.847***
	Episodic delayed recall	0.588*	0.779**
	Semantic naming	-0.083	0.020
	Semantic comprehension	-0.143	-0.019
AD	Episodic Past	0.450	0.576*
	Semantic Past	0.142	0.649**
	Episodic delayed recall	0.128	0.166
	Semantic naming	0.146	0.339
	Semantic comprehension	0.060	0.591*

*p < 0.05; **p < 0.01; ***p < 0.0001.

In bvFTD, episodic future thinking performance correlated strongly with episodic (*p* = 0.003) and semantic (*p* = 0.008) retrieval from the past. Similarly, semantic future thinking performance was significantly associated with the retrieval of episodic (*p* = 0.011) and semantic (*p* < 0.0001) past information.

In contrast, for AD patients, no significant associations were found between episodic future thinking and episodic (*p* = 0.092) or semantic (*p* = 0.613) past retrieval. Semantic future thinking, however, was associated with retrieval of both episodic (*p* = 0.025) and semantic (*p* = 0.009) information from the past.

No significant associations were found between past retrieval and future thinking performance across episodic and semantic domains in the Control group (all *p*-values >0.09).

Secondly, we investigated the relationship between future thinking capacity and performance on neuropsychological tests of episodic and semantic memory. In bvFTD, significant associations were found between episodic future thinking and delayed episodic memory retrieval (RAVLT: *p* = 0.021). Similarly, semantic future thinking was found to correlate with delayed episodic retrieval (RAVLT: *p* = 0.001).

In AD, no significant correlations were found between episodic future thinking performance and neuropsychological tests of episodic and semantic memory (all *p*-values > 0.1), however, semantic future thinking was found to correlate with semantic comprehension (*p* = 0.020).

No significant associations were found in the Control group (all *p*-values >0.1).

### Analyses of Covariance

Based on these correlations, and given the fact that both patient groups showed significant episodic and semantic processing deficits, analyses of covariance (ANCOVA) were run to investigate the effect of general episodic and semantic memory processes on episodic and semantic future thinking, respectively. Age was also included as a covariate in these analyses.

For episodic future thinking, covarying for general episodic memory performance (RAVLT delayed recall) served to ameliorate the overall group effect [*F*(2,41) = 1.211, *p* = 0.308] bringing AD (*p* = 0.651) and bvFTD (*p* = 0.336) patients in line with Control performance. In contrast, controlling for semantic memory performance (SydBAT Comprehension) failed to ameliorate the group effect [*F*(2,43) = 8.102, *p* = 0.001] with both AD (*p* = 0.002) and bvFTD (*p* = 0.003) continuing to show significant impairments relative to Controls.

For semantic future thinking, covarying for semantic processing (SydBAT Comprehension) failed to negate the significant overall group effect [*F*(2,43) = 9.650, *p* < 0.0001] with both bvFTD (*p* < 0.0001) and AD (*p* = 0.006) patients continuing to perform significantly worse than Controls. Interestingly, when we covaried for episodic memory performance (RAVLT delayed recall), semantic future thinking deficits improved in AD (*p* = 0.739) but not in bvFTD (*p* = 0.040) relative to Controls. Finally, controlling for both episodic and semantic memory processes served to ameliorate the semantic future thinking deficit across both patient groups (bvFTD: *p* = 0.065; AD: *p* = 0.816).

### Voxel-Based Morphometry Analysis

#### Patterns of Gray Matter Atrophy

**Table 3** displays the patterns of gray matter intensity decrease in AD and bvFTD participants relative to Controls. AD patients showed widespread neural atrophy across medial temporal, frontal, parietal, and occipital regions of the brain compared to Controls. Significant atrophy was present in right lateral and medial prefrontal cortices, bilateral temporal cortices and temporal poles, medial temporal structures including the bilateral hippocampus, and posterior regions including the left supramarginal and angular gyri, lateral occipital cortices and occipital poles.

**Table 3:** VBM results showing regions of significant gray matter intensity decrease in AD and bvFTD patients relative to controls.

Contrast	Regions	Side	Number of voxels	MNI coordinates		
				*x*	*y*	*z*
AD vs. Controls	Temporal pole, temporal fusiform cortex, OFC, parahippocampal cortex, amygdala, hippocampus, thalamus, insular cortex, parietal operculum cortex, supramarginal gyrus, angular gyrus, occipital cortex, precuneus cortex	L	9,713	-28	4	-48
	Inferior/middle frontal gyrus, insular cortex, paracingulate gyrus, anterior cingulate cortex, posterior cingulate cortex	L	2,720	-40	14	22
	Lateral occipital cortex	R	2,166	46	-86	6
	Paracingulate gyrus, medial PFC, anterior cingulate cortex	R	1,588	14	48	-4
	Frontal pole, medial PFC, OFC	L	452	-14	56	-10
	Temporal pole, temporal fusiform cortex	R	318	34	6	-42
	Cerebellum	L	199	-46	-56	-36
	Juxtapositional lobule, superior frontal gyrus	L	142	-4	4	72
	Hippocampus, amygdala	R	107	26	-12	-16
bvFTD vs. Controls	Cerebellum extending into temporal fusiform cortex, temporal pole, parahippocampal cortex, amygdala, hippocampus, insular cortex, OFC, medial PFC, anterior cingulate cortex, paracingulate gyrus, frontal pole	B	38,955	-32	-76	-58
	Cerebellum	R	5,183	50	-58	-50
	Angular gyrus, lateral occipital cortex	R	1,050	48	-54	20
	Precentral gyrus, middle frontal gyrus	R	463	44	-2	38
	Supramarginal gyrus, superior temporal gyrus	L	402	-48	-44	10
	Cerebellum	R	291	6	-46	-22
	Middle frontal gyrus	R	114	46	14	52
	Precentral gyrus	L	100	-18	-28	42

*MRI scan not available for one Control participant. Age included as a covariate in all contrasts. All clusters reported using the threshold free cluster enhancement method (tfce) and corrected for Family Wise Error (FWE) at *p* < 0.05. For brevity, only clusters above 100 contiguous voxels are reported. All clusters reported *t* > 1.70. OFC, orbitofrontal cortex; PFC, prefrontal cortex; L, left; R, right; B, bilateral; MNI, Montreal Neurological Institute.*

BvFTD patients displayed characteristic gray matter intensity loss predominantly across frontoinsular and medial prefrontal regions, including the anterior cingulate cortex and orbitofrontal cortex, as well as lateral and medial temporal regions including the hippocampus, amygdala, and thalamus, bilaterally. Posterior regions were also significantly affected including the left supramarginal gyrus, right angular gyrus, and the right lateral occipital cortex. These patterns of atrophy are consistent with previous reports in AD (Karas et al., 2004) and bvFTD (Rosen et al., 2002).

#### Neural Correlates of Episodic Future Thinking Performance

**FIGURE 2:**
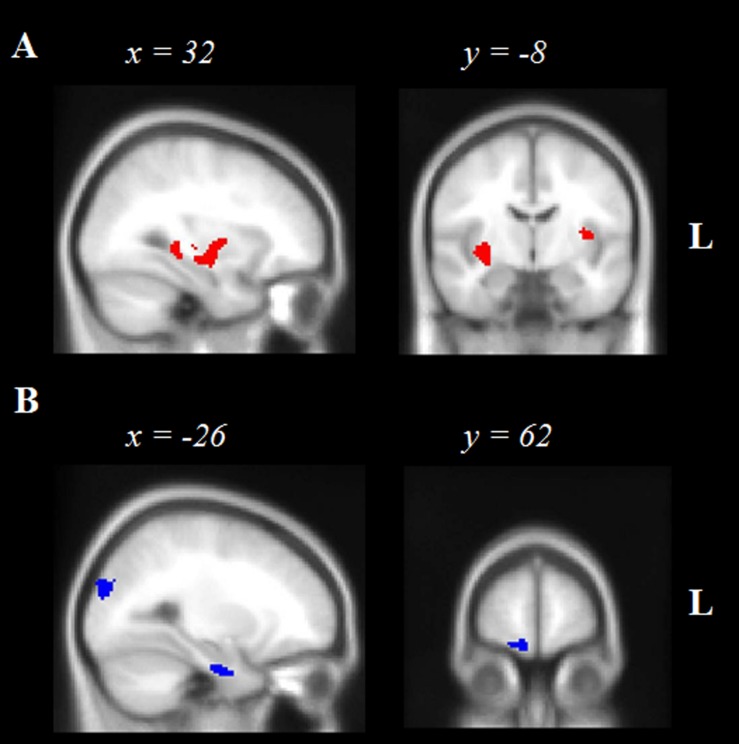
**Voxel-based morphometry results showing brain regions in which gray matter intensity correlates significantly with **(A)** Episodic Future thinking controlling for Episodic Past retrieval, and **(B)** Semantic Future thinking controlling for Semantic Past retrieval in AD and bvFTD participants combined.** Age is included as a nuisance variable in all contrasts. Colored voxels show regions that were significant in the analyses and extracted using a voxelwise approach, corrected for False Discovery Rate at *p* < 0.05. All clusters reported *t* > 3.4 and depict a positive association between gray matter integrity and future thinking performance. Clusters are overlaid on the Montreal Neurological Institute standard brain. L, Left.

**Figure 2A** and **Table 4** display the significant regions to emerge from the covariate analysis investigating the neural correlates of episodic future thinking performance controlling for episodic past retrieval and age. Key regions to emerge included right subcortical structures notably the putamen, amygdala and posterior hippocampus, left temporal regions including the planum temporale and left superior temporal gyrus, the bilateral insular cortices, the left occipital fusiform gyrus, and left cerebellum.

**Table 4:** Voxel-based morphometry results showing regions of significant gray matter intensity decrease exclusively associated with future thinking performance in AD and bvFTD participants (*n* = 30).

Contrast	Regions	Side	Number of voxels	MNI coordinates		
				*x*	*y*	*z*
**Episodic future thinking**						
	Putamen, amygdala, insular cortex	R	246	30	-14	-10
	Hippocampus (posterior)	R	156	36	-32	-6
	Planum temporale, thalamus	L	143	-32	-36	4
	Lingual gyrus	L	141	-6	-84	-20
	Cerebellum	L	120	-20	-58	-50
	Superior temporal gyrus, planum temporale, insular cortex	L	107	-42	-28	-2
	Occipital fusiform gyrus	L	62	-28	-72	-8
**Semantic future thinking**						
	Lateral occipital cortex	R	176	46	-86	6
	Occipital pole, cuneal cortex	R	150	16	-90	18
	Paracingulate gyrus, superior frontal gyrus	L	148	-14	14	46
	Lateral occipital cortex	L	145	-54	-72	-4
	Occipital pole	L	141	-20	-94	28
	Postcentral gyrus	R	91	28	-38	70
	Temporal fusiform cortex, inferior temporal gyrus	L	88	-34	-30	-26
	Inferior/middle temporal gyrus	R	78	50	-28	-18
	Temporal fusiform cortex	R	67	30	-16	-46
	Central opercular cortex, frontal operculum cortex	L	59	-42	4	6
	Parahippocampal cortex, hippocampus	L	56	-24	-6	-32
	Inferior temporal gyrus	R	56	52	-6	-36
	Frontal pole	R	54	20	60	-18

*All clusters extracted using voxelwise approach and corrected for False Discovery Rate at *p* < 0.05. For Episodic future thinking contrast, episodic past retrieval and age are included as covariates. For Semantic future thinking contrast, semantic past retrieval and age are included as covariates. All clusters reported at *t* > 3.4. L, left; R, right; MNI, Montreal Neurological Institute.*

#### Neural Correlates of Semantic Future Thinking Performance

**Figure 2B** and **Table 4** display the significant regions to emerge from the covariate analysis investigating the neural correlates of semantic future thinking controlling for semantic past retrieval and age. Integrity of the bilateral occipital cortices, left prefrontal cortex and right frontal pole, bilateral lateral temporal regions, and left anterior hippocampus was significantly associated with semantic prospection.

## Discussion

To date, investigations of mental time travel have paid disproportionate attention to episodic expressions of future thinking, to the neglect of non-episodic forms. This study investigated the capacity for personal and non-personal forms of future-oriented thinking in the dementia syndromes of AD and the behavioral-variant of frontotemporal dementia. Both patient groups showed striking deficits in future thinking, manifesting across episodic and semantic domains. Analyses of covariance revealed that while episodic future thinking deficits could be explained in terms of episodic memory proficiency, semantic prospection deficits reflected the interplay between episodic and semantic processing impairments. VBM analyses revealed significant associations between MTL integrity and future thinking performance, with the suggestion of lateralization effects contingent on the type of simulation. In addition, differential extra-MTL involvement was observed across episodic and semantic domains. Our findings complement the extant functional neuroimaging literature in healthy individuals to suggest a fundamental role for the hippocampus in supporting future-oriented forms of thought across personal and non-personal contexts.

The most important finding to emerge from this study concerns the striking impairment of semantic future thinking in AD and bvFTD dementia syndromes, which cannot be explained solely in terms of compromised semantic retrieval from the past. Indeed, our covariate analyses suggests that prospection in the semantic domain draws upon contributions from both episodic and semantic memory, underscoring the significant interplay between the episodic and semantic memory systems in supporting past and future mental time travel (Irish and Piguet, 2013). Moreover, this finding was supported on the neuroanatomical level with the observation of significant associations between hippocampal integrity and semantic prospection. Our findings corroborate a previous report of impaired semantic prospection in patients with MTL damage (Race et al., 2013) and converge in favor of a central role for the hippocampus across episodic and semantic forms of future thinking, albeit with some laterality effects.

To date, few studies have investigated the neurocognitive mechanisms of semantic forms of prospection, the vast majority of studies focusing almost exclusively on episodic expressions of future thinking (reviewed by Klein, 2013a). It has been suggested that non-personal forms of temporal projection are largely mediated by relatively intact world knowledge (Klein, 2013b). The proposal that semantic future thinking should draw upon the contents of semantic memory from the past is intuitive, and complements the now well established link between past retrieval and future simulation in the episodic domain (Schacter et al., 2012).

What remains unclear, however, is the extent to which the episodic and semantic memory systems cooperate to support semantic forms of future thinking. Considerable interplay exists between episodic and semantic memory (Greenberg and Verfaellie, 2010; Irish and Piguet, 2013), with episodic memory facilitating the retrieval of semantic knowledge (Westmacott and Moscovitch, 2003). It has been suggested that episodic memory may inform semantic prospection by setting boundary conditions for the scope of generalizations that can be made given the relatively unconstrained nature of semantic prospection (Suddendorf and Corballis, 2007). Our results confirm that the interdependency between episodic and semantic memory also endures in the simulation of non-personal future information, reinforcing previous findings in amnesic patients with MTL lesions (Race et al., 2013). Indeed semantic future thinking appears to hinge upon the integrity of both the semantic and episodic memory systems, underscoring the need to consider flexible interactions between these memory systems to support complex forms of prospection (Szpunar et al., 2014).

In support of a fundamental role for episodic memory processes in semantic forms of future thinking, we found significant associations between hippocampal integrity and the capacity to engage in semantic prospection. Our findings resonate strongly with those of Race et al. (2013), who suggested that MTL damage may disrupt semantic prospection in amnesia by impairing generative semantic retrieval and associative binding processes. Simulating a non-personal future likely depends upon many of the fundamental mechanisms that are required for successful prospection in the episodic domain; however, semantic future thinking may recruit additional recombinatorial processes given the undifferentiated nature of conceptual information and the relatively unconstrained and open-ended nature of semantic prospection (Abraham et al., 2008). The hippocampus is crucial for recombinatorial and elaborative processes during novel future event simulation (Addis and Schacter, 2008) and has also been shown to play a fundamental role in supporting non-episodic prospection. Abraham et al. (2008) demonstrated that the hippocampus was recruited when healthy individuals envisaged episodic and semantic future issues, suggestive of a common core substrate supporting both forms of prospection. Notably, however, reaction times were significantly longer during non-personal future trials relative to non-personal past trials, a finding that was interpreted as reflecting a higher degree of constructive and associative processing when recombining existing conceptual elements from semantic memory (see also Suddendorf and Corballis, 2007; Abraham et al., 2008).

The proposal that future-oriented forms of cognition differentially stress hippocampally dependent recombination processes has been made previously with regard to episodic future simulation (Addis et al., 2011). Here, we demonstrate a significant association between hippocampal integrity and episodic and semantic forms of prospection, suggestive of a possible domain-general contribution of this structure in mediating future-oriented forms of thought. The right hippocampus in particular has emerged as a crucial structure in supporting constructive episodic future simulation (Addis et al., 2007, 2011) as well as the construction of spatially integrated atemporal scenes (Mullally et al., 2012, 2014). The fact that our correlation analyses implicated the right hippocampus exclusively for episodic forms of simulation is noteworthy and provides important lesion data to complement findings from the fMRI literature on healthy individuals. The precise contribution of the right hippocampus to future-oriented thinking remains unclear, and it will be important for future studies to disambiguate the role of various candidate mechanisms including generative retrieval, detail recombination, and integration within a coherent framework in this process.

While our findings suggest a central role for the hippocampus in future oriented mental travel, it is clear that memory and prospection deficits in dementia arise in the context of large-scale network disruption (Irish et al., 2012c). As such, impaired capacity for prospection in these patients likely also reflects the degeneration of extra-hippocampal regions, as has been demonstrated for other constructive endeavors including scene construction (Irish et al., 2015). Indeed, our whole-brain VBM analyses revealed significant involvement of insular, lateral temporal and occipital regions in episodic future thinking impairments, resonating with the observation of a distributed set of regions which underpins the capacity for episodic prospection in healthy individuals (Schacter et al., 2012). Where semantic prospection is concerned, we found significant prefrontal, lateral temporal and occipital involvement again in keeping with extant findings in the literature. It is noteworthy that prefrontal contributions, including the superior frontal gyrus and right frontal pole, were found exclusively in the semantic prospection condition. Previous studies have posited that activation of the superior frontal gyrus may reflect greater demands on generativity, verbal fluency, and flexibility (Abraham et al., 2008), while others have proposed that frontopolar activation on simulation tasks may reflect greater constructive demands inherent to future simulation (Schacter et al., 2007) or the representation of the temporal component of future episodes (Okuda et al., 2003). The precise contribution of prefrontal regions to prospection remains unclear, and as such, it will be important for future studies to tease apart the relative contribution of prefrontal versus medial temporal regions to complex expressions of future thinking (see for example Dermody et al., 2015).

A number of methodological limitations warrant consideration. Firstly, while we have demonstrated that semantic prospection is significantly compromised in these dementia syndromes, the underlying mechanisms mediating these deficits are likely to be multifactorial. Relative to previous studies, the task used here is arguably much simpler in that elaboration is not required; rather, participants are asked to simply generate exemplars of past and future personal and non-personal issues. This parsed down approach was necessary, however, to circumvent some of the potential challenges inherent in testing patients with dementia, such as apathy and fatigue. Secondly, while general prompts were provided to participants during the experimental task, it is possible that the past and future thinking deficits observed here reflect more general difficulties with strategic retrieval in light of significant prefrontal atrophy in these syndromes. We believe this explanation is unlikely, however, as no significant correlations were found between neuropsychological measures of working memory or verbal fluency and the experimental task. Thirdly, our patients were in the moderate stages of the disease and as such it remains unclear at what stage of the pathological process difficulties with semantic prospection emerge. To address this issue, we suggest that studies targeting the prodromal syndrome of Mild Cognitive Impairment, characterized by relatively circumscribed MTL pathology, will serve to clarify the neurocognitive mechanisms underpinning semantic future thinking deficits in dementia.

## Conclusion

This study demonstrates that future thinking deficits extend to the semantic domain in patients with AD and the behavioral-variant of frontotemporal dementia, attributable to semantic and episodic processing impairments. We suggest that semantic prospection reflects a confluence of episodic and semantic processes, drawing upon a distributed set of prefrontal, lateral and medial temporal regions in the service of generative retrieval and associative binding. Future studies investigating the precise contribution of medial temporal and frontopolar regions to non-episodic forms of prospection will be important to further advance our understanding of how humans engage in sophisticated acts of future-oriented mental time travel across personal and non-personal domains.

## Author Contributions

MI was responsible for study conception and design, data acquisition, data analyses and interpretation, writing of the manuscript, and agreeing to be accountable for all aspects of the work. NE was responsible for data acquisition, data analyses and interpretation, drafting of the work, and agreeing to be accountable for all aspects of the work. ND was responsible for data acquisition, data analyses and interpretation, drafting of the work, and agreeing to be accountable for all aspects of the work. CO was responsible for study conception and design, data acquisition, revising the manuscript critically for important intellectual content, and agreeing to be accountable for all aspects of the work. JRH was responsible for study conception and design, data interpretation, revising the manuscript critically for important intellectual content, and agreeing to be accountable for all aspects of the work. MH was responsible for study conception and design, data interpretation, revising the manuscript critically for important intellectual content, and agreeing to be accountable for all aspects of the work. OP was responsible for study conception and design, data interpretation, revising the manuscript critically for important intellectual content, and agreeing to be accountable for all aspects of the work.

## Conflict of Interest Statement

The authors declare that the research was conducted in the absence of any commercial or financial relationships that could be construed as a potential conflict of interest.

## Footnotes

1 http://www.fmrib.ox.ac.uk/fsl/fslvbm
